# On the phenology of soil organisms: Current knowledge and future steps

**DOI:** 10.1002/ece3.10022

**Published:** 2023-04-25

**Authors:** Ana E. Bonato Asato, Christian Wirth, Nico Eisenhauer, Jes Hines

**Affiliations:** ^1^ German Centre for Integrative Biodiversity Research (iDiv) Halle‐Jena‐Leipzig Leipzig Germany; ^2^ Institute of Biology Leipzig University Leipzig Germany

**Keywords:** fauna, seasonality, soil activity, soil biodiversity, soil biomass, temporal patterns

## Abstract

Phenology is the study of timing of periodic activities in biological life cycles. It describes an inherent component of ecosystem dynamics, and shifts in biological activity have been increasingly recognized as an indicator of global change. Although phenology is mainly studied above the ground, major ecosystem processes, such as decomposition, mineralization, and nutrient cycling, are soil‐dependent. Therefore, the phenology of soil organisms is a crucial, but understudied, aspect of terrestrial ecosystem functioning. We performed a systematic review of 96 studies, which reported 228 phenological observations, to evaluate the current knowledge of soil microbial and animal phenology. Despite the increasing number of soil phenology reports, most research is still concentrated in a few countries (centered in the Northern Hemisphere) and taxa (microbiota), with significant gaps in the most diverse regions of the globe (i.e., tropics) and important taxa (e.g., ants, termites, and earthworms). Moreover, biotic predictors (e.g., biodiversity and species interactions) have rarely been considered as possible drivers of soil organisms' phenology. We present recommendations for future soil phenology research based on an evaluation of the reported geographical, taxonomic, and methodologic trends that bias current soil phenology research. First, we highlight papers that depict good soil phenology practice, either regarding the research foci, methodological approaches, or results reporting. Then, we discuss the gaps, challenges, and opportunities for future research. Overall, we advocate that focusing both on highly diverse ecosystems and key soil organisms, while testing for the direct and indirect effects of biodiversity loss and climatic stressors, could increase our knowledge of soil functioning and enhance the accuracy of predictions depicting the effects of global change on terrestrial ecosystem functioning as a whole.

## Graphical abstract

Although phenology is mainly studied in plants, major ecosystem processes are soil‐dependent, indicating that the phenology of soil organisms is a crucial aspect for the functioning of terrestrial ecosystems. Previous work have identified the importance of taking into account the multidimensionality of soil (e.g., geographical, depth, and temporal variation), but no work has built on the best ways of doing it. Through a systematic review, we identify the main biases and gaps in soil phenology research and build a list of recommendations for future research, to improve efficiency in sampling and realism of phenological patterns detection. Most importantly, we advocate for the construction of collaborative networks between above‐ and belowground experiments towards a more comprehensive understanding of the total environment, especially in a changing world.

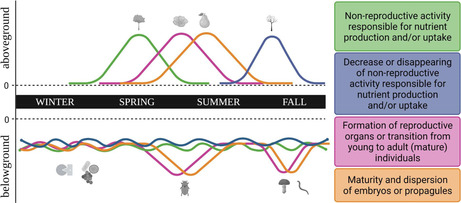

## 1 INTRODUCTION

The study of annually recurring biological activities in relation to climate, known as phenology, is a long‐standing field of science that has now regained attention due to its importance for ecosystem stability (Valencia et al., 2020) and ecosystem responses to global change (Cleland et al., 2006; Cotton, 2003; Körner & Basler, 2010; Parmesan & Yohe, 2003). Cycles of biological activities have been reported across multiple taxa and biomes (Mellard et al., 2019). However, plant studies dominate phenological research due to the relative ease of studying visible aboveground plant tissues and plants' primacy as primary producers in determining the timing of many ecological processes (e.g., decomposition, mineralization, and nutrient cycling) (Chen & Coops, 2009; Cuevas & Medina, 1985). Nevertheless, many processes are tightly tied to the soil compartment in terrestrial ecosystems. Therefore, from microscopic organisms to large earthworms, soil organisms are likely to co‐determine the timing of multiple ecosystem processes (Bardgett & Wardle, 2010). This suggests that examining the phenology of soil organisms is essential to better understand how terrestrial ecosystems function (Eisenhauer et al., 2018), making soil phenology a high‐priority topic in ecological research (Eisenhauer et al., 2017).

At least three subjects suggest that soil phenology demands attention that is distinct from aboveground phenology. These are related to (1) aboveground‐belowground mismatches in activity patterns; (2) global biodiversity distribution; and (3) the unknown role of biotic interactions. First, belowground organisms may have different strategies to deal with climatic perturbations than what we know from tissues and organisms in the aboveground compartment (i.e., plant shoots and aboveground animals). Environmental conditions, such as temperature and moisture, may be distinctly different aboveground versus belowground. Therefore, even when living in close spatial proximity, organisms on opposite sides of the aboveground‐belowground spectrum may be facing different metabolic constraints (Decaëns, 2010; Wu et al., 2011). For example, the environment can impose substantial seasonal variation on the activity of plants and aboveground animals, but the activity of soil fauna and soil microbes (hereafter soil activity) is more uniform due to the thermal buffering capacity of the soil or a wider range of tolerance of soil organisms. This may be particularly pronounced in harsh environments, such as cold deserts and semiarid or arid ecosystems, where plants exhibit a dormant period, maintaining only basal aboveground activity levels during periods of cold or dry weather (Abramoff & Finzi, 2015). The belowground compartment (i.e., plant roots, soil microorganisms, and animals) may be more developed in these ecosystems. A more complex root system and associated microbes and fauna could uncouple above‐ and belowground phenological patterns (Blume‐Werry et al., 2016). For example, the growing season for plant roots is longer than that of plant shoots in the Arctic (Blume‐Werry et al., 2016). With the extended root activity, soil microbiota, and fauna can be active for longer periods, making soil phenology less seasonal or rather a different timing of the seasonality than aboveground phenology.

Second, differences in phenology may be underpinned by greater temporal niche partitioning in more diverse communities (Hooper, 1998). However, biodiversity may have dissimilar distribution above and below the ground (Bastida et al., 2020; Fierer et al., 2009; Gaston, 2000; Phillips et al., 2019). Although climatic factors like temperature and rainfall drive both aboveground and belowground diversity patterns, global distribution patterns of belowground communities differ from those seen in aboveground organisms (Bardgett & van der Putten, 2014; Cameron et al., 2019; Gaston, 2000). For example, most fungal groups and some amoebae exhibit greater local diversity in low latitude, tropical regions (Lara et al., 2016; Tedersoo et al., 2014), but ectomycorrhizal fungi and earthworms show greater local diversity in high latitudes (Phillips et al., 2019; Tedersoo et al., 2014). Due to uncertainties in the relationship between latitude and soil biodiversity, it has been proposed that above‐ and belowground distributions are driven by different mechanisms (Bardgett & van der Putten, 2014; Gaston, 2000). This raises the question of whether areas with marked mismatches between above‐ and belowground diversity patterns (Cameron et al., 2019), or areas with high plant seasonality, also tend to show more decoupled above‐ and belowground phenology. The high belowground diversity may support a wider range of phenological niches, which may lead to a more homogeneous activity pattern throughout the year. If that is true, a decoupled phenology may be observed between plants and soils, in ecosystems of seasonal plant activity.

Third, species interactions have been recognized as key drivers of phenology. However, soil phenology research has not yet accounted for interactions among soil animals. From aboveground studies on plants, species competition and facilitation can be critical in determining the timing of crucial aboveground phenological events (Camargo et al., 2013; Kochmer & Handel, 1986). For example, plant species richness is known to increase the species richness of pollinators, and competition strength among pollinators determines how plant species will partition temporally available resources needed to grow and reproduce more efficiently (Elzinga et al., 2007; van Schaik et al., 2003; Wheelwright, 1985). We currently have minimal information regarding if and how biotic interactions affect soil phenological patterns.

In this review, we first assess the features of soil phenology research to date by identifying geographical and taxonomical foci and biases. We discuss where, when, and why the biases may (or may not) be matched with the geographic distribution of species and strong phenological variation. Based on the literature reviewed, we point out papers that have proposed questions and practices that pave the way for a new research field, or that substantially advanced soil phenology research towards solving needs we currently have with regards to the questions asked, methodological approach, or any other feature that may characterize novelty. Finally, we identify the main gaps, challenges, and research opportunities to inspire future research that will improve our ability to make clear recommendations for land management and policy decisions. Taken together, soil phenology should strive to clarify the variation in temporal patterns across places and taxa and establish clear relationships between these temporal patterns and biotic, as well as abiotic drivers. Only then will shifts in soil phenology be helpful in indicating and predicting whether and how biological systems will be affected by global change factors.

## 2 METHODS

### 2.1 Data collection

We searched for papers published online through Clarivate Analytics Web of Science core collection with the following combination of search terms in their titles or abstracts: (“phenolog*” OR “temporal”) AND (“dynamic*” OR “activity” OR “pattern*”) AND (“belowground*” OR “soil” OR “edaphic*”) AND (“soil biot*” OR “soil org*” OR “soil micro*” OR “soil macro*” OR “soil meso*” OR “soil animal*” OR “soil arthropod*” OR “soil invert*” OR “soil fauna*” OR “soil divers*” OR “soil biodivers*” OR “soil fung*” OR “soil bact*” OR “mycorrhiza*”). We refined the search by looking at the following categories: ecology, environmental sciences, plant sciences, atmospheric meteorology sciences, geosciences multidisciplinary, agronomy, remote sensing, multidisciplinary sciences, forestry, entomology, zoology, biodiversity conservation, geography physical, geochemistry geophysics, evolutionary biology, soil science, biology, agriculture multidisciplinary, microbiology, biotechnology applied microbiology, environmental studies, toxicology, and agricultural engineering. Gray literature (books and book chapters) and reviews were excluded, since they are either based on published papers that are eligible to be encompassed by the search. That search returned 6427 papers on the June 1, 2020. We screened the titles and abstracts of all papers, looking for those matching broadly with the topic, which means they (i) were carried out in terrestrial ecosystems and (ii) reported soil biodiversity, abundance, biomass, and/or activity as a response variable. This resulted in a list of 243 papers. After reading these selected publications in full, we kept those empirical studies, which (iii) conducted at least four measurements in a year. A few papers were also added later, after suggestions by reviewers. In total, we extracted information from 96 papers. Because individual papers often explore more than one phenological response, we accounted for 228 phenological observations (see Table S1).

### 2.2 A short glossary of the information recorded

For each of the 228 phenological observations, we extracted the following information:

1. Country and continent where the study was conducted were recorded in order to build a world map of soil phenology research, and identify well‐ and under‐represented areas.
2. Study type was classified as “experimental” when one or more of the predictor variables were directly manipulated or as “observational” when no intervention nor manipulation of variables was made.
3. Climatic zone where the study was conducted was classified according to the latitude. Tropical zones encompassed areas between 25° N and 25° S; subtropics ranged from >25° to 40°; temperate zones ranged from 40° to 60°; and every place >60° was classified as a polar zone.
4. Ecosystem type was classified as grasslands, forests, or deserts. Because of the limited occurrence of other ecosystem types, everything else (e.g., shrublands, dunes) was classified as “other.”
5. Land‐use type was classified as agricultural, pastural, natural, or recreational land (e.g., parks or other urbanized places), based on information provided by the papers.
6. To assess the most often used sampling practices, we extracted the sampling duration, as the interval between the first and the last sampling, the total number of samplings, and the sampling interval. When conducted regularly, the sampling interval was classified as “up to monthly” when the interval between two sampling events was less than 1 month, as “monthly” for monthly samplings, and as “> monthly” for intervals longer than 1 month. When the interval between samplings did not follow any clear pattern, the sampling interval was classified as “irregular.”
7. Soil depth was classified according to the deepest depth where the measurement was made (e.g., soil cores taken from the litter layer until 15‐cm deep were classified as “15 cm”). For measurements made at the litter layer (e.g., soil respiration), the depth was zero.
8. Because soil biodiversity is high (Giller, 1996), and different types of organisms may respond differently to biotic and abiotic constraints, we extracted information on soil organism type, as classified following the Global Soil Biodiversity Atlas classification (Orgiazzi et al., 2016). In this way, macrofauna mainly includes macroarthropods, such as large insects (e.g., ants, beetles) and Arachnida (e.g., spiders and scorpions), as well as gastropods and earthworms. Mesofauna covers Tardigrada and microarthropods, such as Collembola and mites. Microbiota encompasses the microbiota, such as Protozoa, Nematoda, Rotifera, and the “microflora.” such as bacteria and fungi. Because we did not find any study on megafauna groups (e.g., voles, moles), they could not be included. More specific taxonomic information, such as species and other taxon names, was extracted whenever possible and is available in Table S1.
9. Each phenological observation was classified according to the type of response variable. Because phenology below the ground does not necessarily correspond to any analog above the ground (Box 1), we accounted for responses that are not commonly included in classic aboveground phenology studies. For example, abundance and biomass were used to quantify temporal variation in the number of individuals/area and mass/area over a year, respectively. Seasonal changes in biodiversity were reported as the temporal variation of the number of species (i.e., richness) or any other diversity metric (e.g., Simpson's and Shannon's, trait and phylogenetic diversity). Finally, temporal variations reflecting metabolic and behavioral aspects (e.g., respiration) were classified as “activity.”

BOX 1 **The analogies and dissimilarities between phenology above and below the ground.**

**FIGURE 1:**
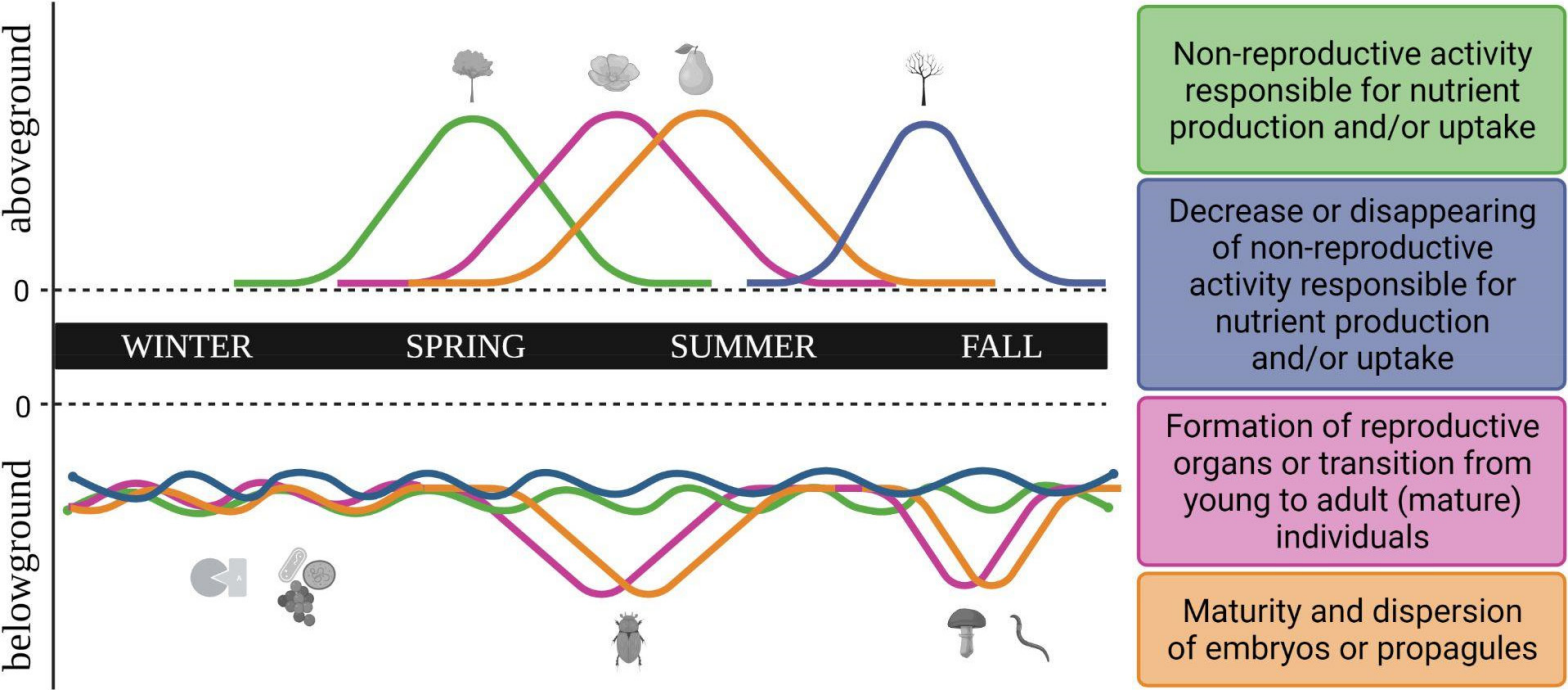
Broad patterns in the phenology of plant and soil organisms in a temperate forest. Using the climatic patterns of a temperate ecosystem, summarized in the x‐axis by the four seasons, the conceptual graph indicates broad phenological patterns of plants and soil organisms above and below the ground. Similarities and dissimilarities between aboveground and belowground subsystems are shown as differences in the timing and magnitude of peaks on the y‐axis. While the magnitude of aboveground phenological activities always ceases (i.e., reaches the zero line), soil organisms and functions are almost always active, with the exception of extreme drought or winter, when soil activity also declines substantially.

Aboveground phenology is usually measured by responses with clear beginnings and ends, such as the appearance of determinate organs or the start of specific activities in an individual, and can then be summarized for the population. Such clear assignments may be challenging below the ground. For example, flowering phenology is classically studied by e.g., counting the number of flowers and their exact development stage in an individual plant, or part of a plant. This approach is not feasible below the ground, since tracking an individual's offspring for most soil organisms (e.g., invertebrates in general and many fungi) under field conditions is challenging. The shape of the phenological activity curves below the ground also hampers the usage of single events commonly reported in plant phenology (e.g., first flowering shown as pink lines aboveground in Box 1, Figure 1). Analogous events below the ground (e.g., reproduction of hypothetic soil microfauna depicted as pink lines belowground Box 1, Figure 1) happen nearly continuously, with some changes in their magnitude driven by specific life histories of different organisms (Box 1, Figure 1). Soil phenological patterns can be best visualized as population, or community‐level responses, or by measures of ecosystem functions.

### 2.3 Qualitative analysis describing the evidence

Due to the wide variety of spatial scales, sampling designs, statistical methods, and other aspects among the reviewed studies, it was not feasible to perform a quantitative meta‐analysis to assess abiotic and biotic drivers affecting soil phenology. Therefore, we qualitatively assess the available information based on the direction of the effects reported for each suitable relationship found in the reviewed studies. A suitable relationship was defined here as a direct relationship between a driver and a phenological observation, in which both were paired temporally. In this way, if a given paper reported the effects of different crop varieties on the phenology of beetles, this relationship was not included since crop variety does not change temporally. We then categorized all suitable relationships between the driver and soil phenology as positive, neutral, or negative. Relationships were classified as “neutral,” as a synonym of “no effect,” when *p*‐values exceeded 0.05. We considered relationships quantified as both correlations and regressions.

## 3 CURRENT KNOWLEDGE OF SOIL PHENOLOGY

Research focusing on soil phenology is gaining momentum but still rare. From the revised pool of papers, the number of publications on soil phenology before the 2000s was around 5 per year. From 2000 to 2010, this number increased to 10, and more recently it is about 20 publications per year. However, despite the increase in the absolute number of publications on soil phenology, it may not represent a real increase of interest in the topic, given that overall increases in scientific publications and publications on plant phenology may indicate a proportional decrease in the number of soil phenology publications.

### 3.1 Where?—Geographical biases of soil phenology research

**FIGURE 2:**
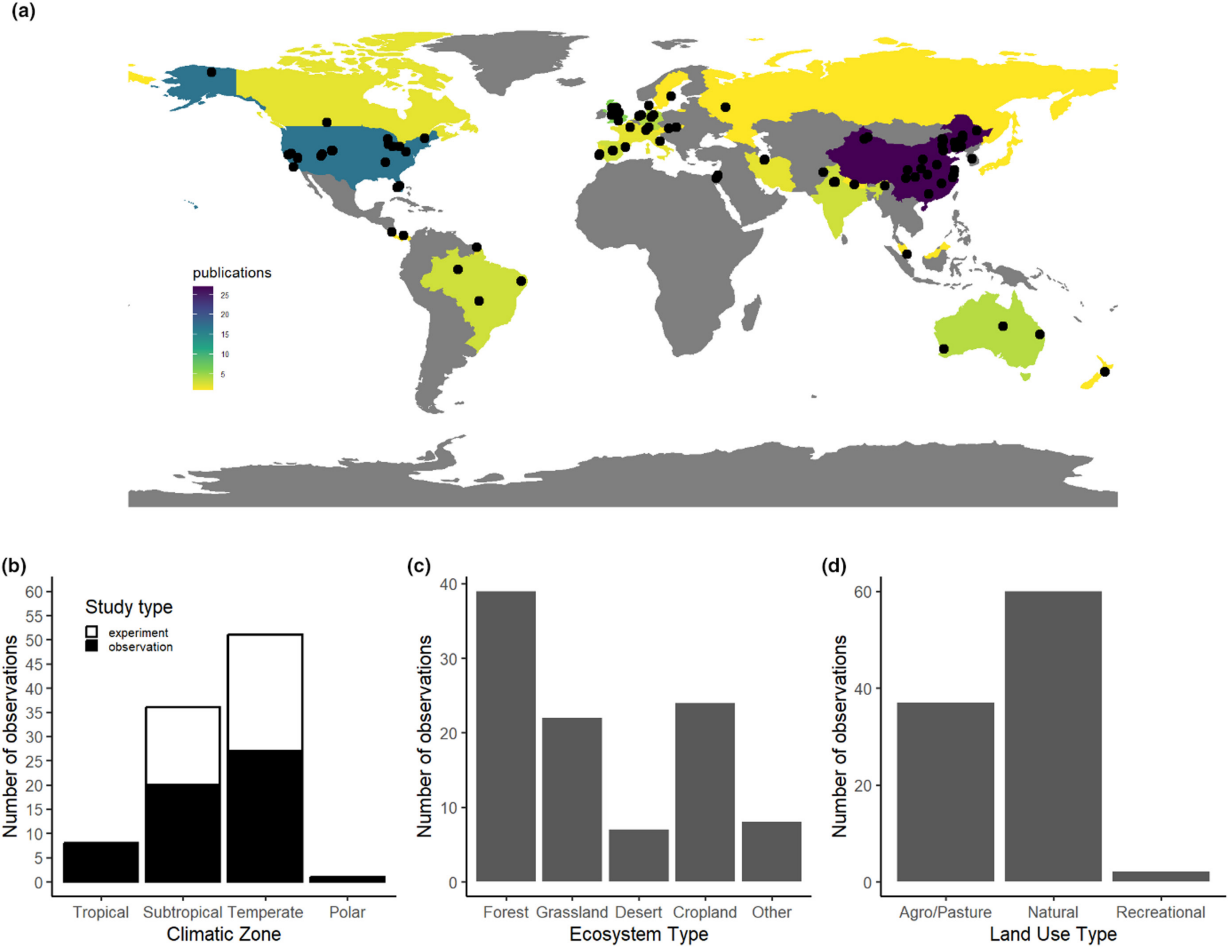
Temporal and geographical trends of soil phenology research. (a) The number of publications on soil phenology conducted by country highlights a scientific bias to the Global North. The black points indicate where exactly each study was conducted, following the coordinates given by the authors. When many coordinates were given, but the results referred to all of them, we used the average values as coordinates. In cases where only a general location was given (e.g., name of a park or city), we defined the coordinates as the center of the location. Five papers did not give enough information to approximate the location and were not represented by a point on the map. The gray areas represent countries without any study on soil phenology. (b) The number of phenological observations by climatic zone, according to the study type (i.e., experiment or observation). (c) The number of phenological observations by ecosystem type, showing that the majority of the studies are concentrated in forests, croplands, and grasslands. (d) The number of phenological observations by land‐use type highlights that temporal measurements are often done in natural and in agricultural lands and pastures.

The soil phenology research that fit our search criteria was conducted in 30 countries. The majority of studies were carried out in the Northern Hemisphere, especially in Asia (99 observations), Europe (78 observations), and America (43 observations). The United States (17 observations) and China (27 observations) had the highest number of studies. Soil phenology was less frequently studied in South and Central Americas and Oceania, while Africa was not represented at all in the soil phenology literature (Figure 2b). As a result, soil phenology in temperate and subtropical zones was comparatively well‐studied. Tropical ecosystems followed, but the difference in the number of studies between the different climatic zones was large (119, 99, and 9 observations in temperate, subtropical, and tropical zones, respectively, Figure 2c). We only found one study carried out in the polar zone, besides its high seasonality and high importance from the climate change perspective. The same geographical bias was already pointed out by other global studies on soil biodiversity (Beaumelle et al., 2021; Guerra et al., 2021; Phillips et al., 2019), recognized as a problem to be overcome (Ramirez et al., 2018), and it is seen as a reflection of social, political, and economic inequalities among countries (Maestre & Eisenhauer, 2019).

Forests, croplands, and grasslands were the most represented ecosystems (Figure 2d). Specifically, most forests were subtropical or temperate, and few soil phenology studies were carried out in rainforests and tropical dry forests (Table S1). Soil phenology in grasslands was mostly studied in the United States and Europe, making prairies and steppes relatively well‐represented with regard to natural grasslands worldwide. In addition, many grasslands were also submitted to some degree of pasture, which explain the higher number of agro/pasture observation (Figure 2e) than pure croplands. Deserts were poorly studied, even though most desert fauna is below the ground as a strategy to survive the harsh temperatures. Other ecosystem types included shrublands and dunes (Table S1).

Most of the studies were observational, and experiments on soil phenology were concentrated in subtropical and temperate ecosystems (Figure 2c). Classically, phenology research is done through long‐term observational studies. Only recently have experiments been used in phenology research. For example, experiments have been used to test the effects of plant diversity and warming on plant phenology (see Guimarães‐Steinicke et al., 2019; Stuble et al., 2021), and have also been used to evaluate plant phenology as a driver of soil processes (as in Fu et al., 2002). Because the set‐up and maintenance of experiments are usually more expensive than observational studies, especially when requiring equipment to control warming and rainfall, the lack of soil phenology experiments in the tropical zones may also be related to the economic inequality between the Global North and South.

Previous reviews have suggested that land‐use type is a major driver of soil phenology (Bardgett & van der Putten, 2014). Most studies considered here were conducted in natural or agricultural lands and pastures (Figure 2e). Because soils play a key role in food production, influencing human well‐being (Wall et al., 2015), further research is needed to understand the role of different crops, crop rotations, and land‐use intensities on soil phenology. Additionally, with the human appropriation of the terrestrial land surface for mixed purposes, and the increasing movement for greening up the cities, further research in recreational areas, mostly in the urban space, is recommended.

### 3.2 What?—The most popular soil organisms and response variables

**FIGURE 3:**
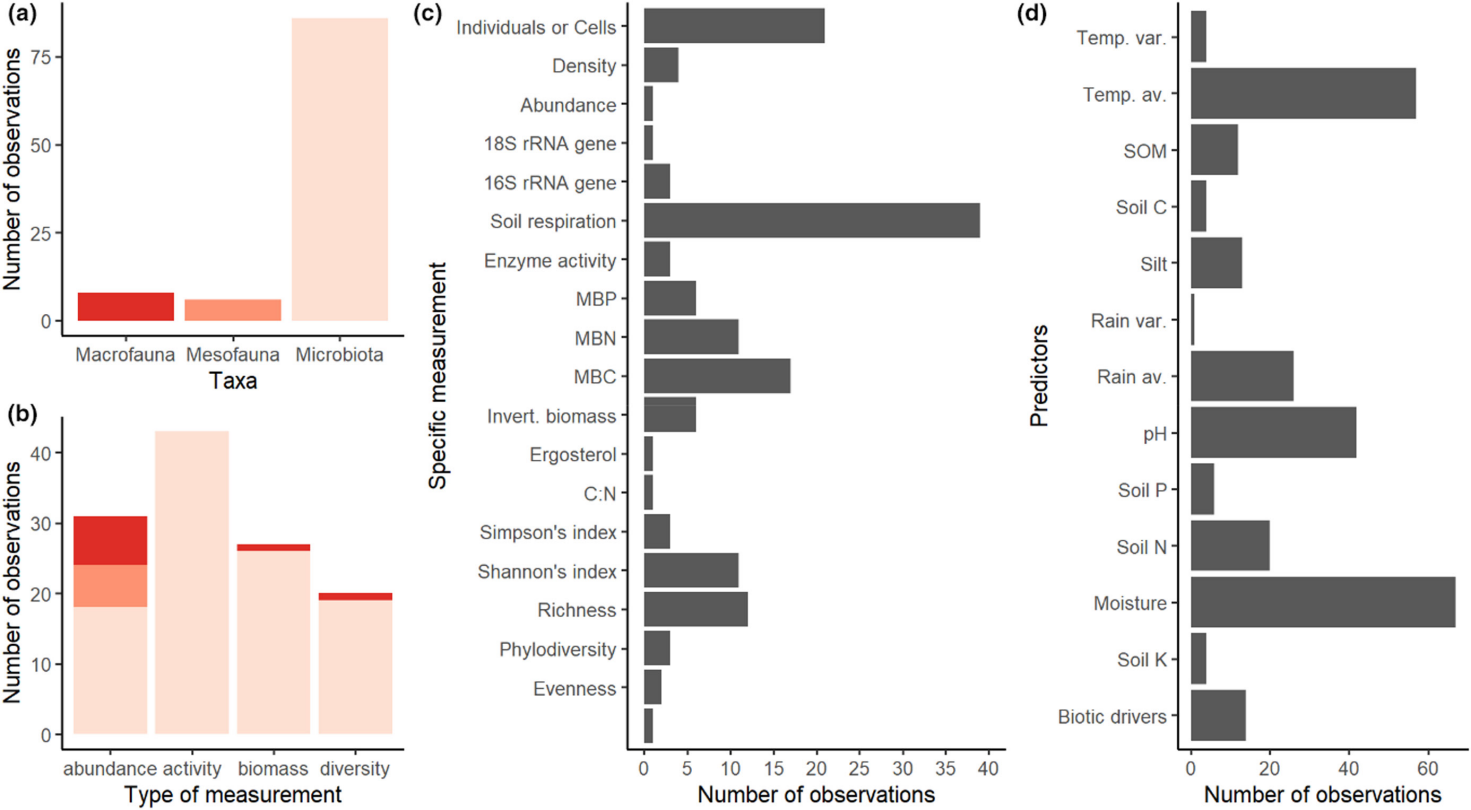
Thematic patterns in soil phenology research. (a) The number of phenological observations by taxa, highlighting a bias towards microbiota. (b) The number of phenological observations by broad type of measurement, colored by taxa as depicted in panel a. (c) The number of phenological observations by specific type of measurement. (d) The number of observations by the predictor, highlighting the disproportional tendency to account for abiotic predictors. C:N, carbon‐to‐nitrogen ratio; Invert. Biomass, invertebrate biomass; MBC, carbon microbial biomass; MBN, nitrogen microbial biomass; MBP, phosphorus microbial biomass; Rain. av., rainfall average; Rain. var., rainfall variation; Soil C, soil carbon (total, organic, or organic); Soil K, soil potassium; Soil P, soil phosphorus; SOM, soil organic matter; Temp. av., soil temperature average; Temp. var., soil temperature variation.

Microbiota was by far the most well‐represented group of organisms in soil phenology studies (86 observations, Figure 3a). Consequently, the phenology of microbial activity and soil microbial biomass were comparatively well‐studied (Figure 3b). Soil respiration was the most studied variable (Figure 3c), which explains the high number of “activity” studies. Many soil respiration studies also measured microbial biomass and/or abundance (Table S1), reflected by the high number of observations of microbial carbon, nitrogen, and phosphorus and the observations on the number of cells (Figure 3c). Besides unicellular organisms, microbiota also included nematodes. However, nematodes represented a small contribution to the number of observations of abundance, biomass, density, and diversity metrics (taxonomic richness, Shannon's diversity, and Simpson's diversity). Although we found studies investigating different aspects of soil phenology that covered a wide range of countries and ecosystem types, the number of nematode studies by phenology type, country, and ecosystem type was meager, precluding any generalization on nematode phenological trends.

Mesofauna phenology was limited to 6 observations that were predominately reported on Collembola, but we also found studies on Acari and tardigrades (Table S1), mostly concentrated on temporal changes in organisms' abundance (Figure 3b). Although recognized as a highly diverse group, studies encompassing the phenological changes in mesofauna diversity were not found. Evidence for turnover (β‐diversity) in mesofauna activity could reflect strong temporal niche partitioning among species. Our lack of evidence may not reflect a lack of effects, but rather a difficulty reaching a high taxonomic resolution, resulting in many undescribed and/or cryptic species (White et al., 2020).

Little attention has been given to soil macrofauna in phenological studies (8 observations), despite its central role in crucial ecosystem processes and contribution to soil health (Ettema & Wardle, 2002; Scheu & Schaefer, 1998). Among the potentially diverse pool of macrofauna taxa, the studies were mainly focused on phenological observations of earthworms and beetles, with one paper addressing one species of ant (Table S1). Together, earthworms and ants are considered ecosystem engineers, and their biological activity is a key driver of community assembly and ecosystem functioning (Bardgett & van der Putten, 2014; Jouquet et al., 2006). However, they differ in their geographical distribution—while earthworm communities are diverse and abundant in the subtropics to temperate ecosystems (Phillips et al., 2019), ants are more abundant and diverse in tropical and subtropical regions, emphasizing arid and semiarid ecosystems, especially for the ants (Crawford, 1991). The discrepancy in the number of studies across taxa may be related to where they mainly occur and play a significant role in the ecosystem since most studies are conducted in subtropical and temperate regions (Figure 2c).

Soil biodiversity covers a wide range of taxa, from microbes to megafauna, encompassing various ecological roles and associated ecosystem processes (Giller, 1996). This diversity may, in turn, lead to variation in phenological responses. Consequently, phenological studies need to evaluate the responses of species with different body sizes, reproductive patterns, and life‐history traits. Moreover, the level of ecological organization captured may also influence the sensitivity of phenological measurements. Although diversity metrics are typically measured at the community level, abundance, and biomass can be measured for populations or communities, and activity can be studied for individuals, populations, or communities (Eisenhauer et al., 2018). Because temporal trends of higher ecological levels (e.g., communities vs. populations) result from the cumulative temporal trends of all lower ecological levels, patterns identified from one level of ecological organization cannot be extrapolated to other resolutions (e.g., from a single population to an entire community or vice versa) (Morin et al., 2014; Xu et al., 2021). Although changes in abundance might reflect changes in population dynamics, we suggest that an overall view of community dynamics will improve predictions of the consequences of global change for ecosystem functioning. Ultimately, improved predictions will provide better decision support for sustainable soil policy worldwide (Guerra et al., 2021).

Most of the studies accounted for abiotic predictors only (Figure 3d). Drivers related to water availability, such as soil moisture and rainfall average, were often associated with soil phenology. The focus on temperature‐related variables, such as average soil temperature and soil temperature variability, were also commonly reported as drivers of biological activity (Davidson et al., 1998; Schwieger et al., 2019; Shi et al., 2020; Song et al., 2015). Soil pH was also often considered, reflecting the importance of acidity in determining soil functioning (Mulder & Elser, 2009). Regarding soil fertility, soil N was the most studied predictor. No study in our search has investigated the effects of rainfall variability on soil phenology (although a few studies have found negative effects of rainfall extremes on soil biota in nonphenological approaches, e.g., Lensing & Wise, 2006, Wise & Lensing, 2019). Importantly, rainfall variability is being focused on in climate change research since rainfall is expected to be increasingly irregular (Konapala et al., 2020). This highlights the need to account for resource heterogeneity variables by manipulating them experimentally (e.g., Dang et al., 2009) or conducting comparative research in ecosystems where they are, and are not, already irregular.

### 3.3 How?—Methodological trends in soil phenology research

**FIGURE 4:**
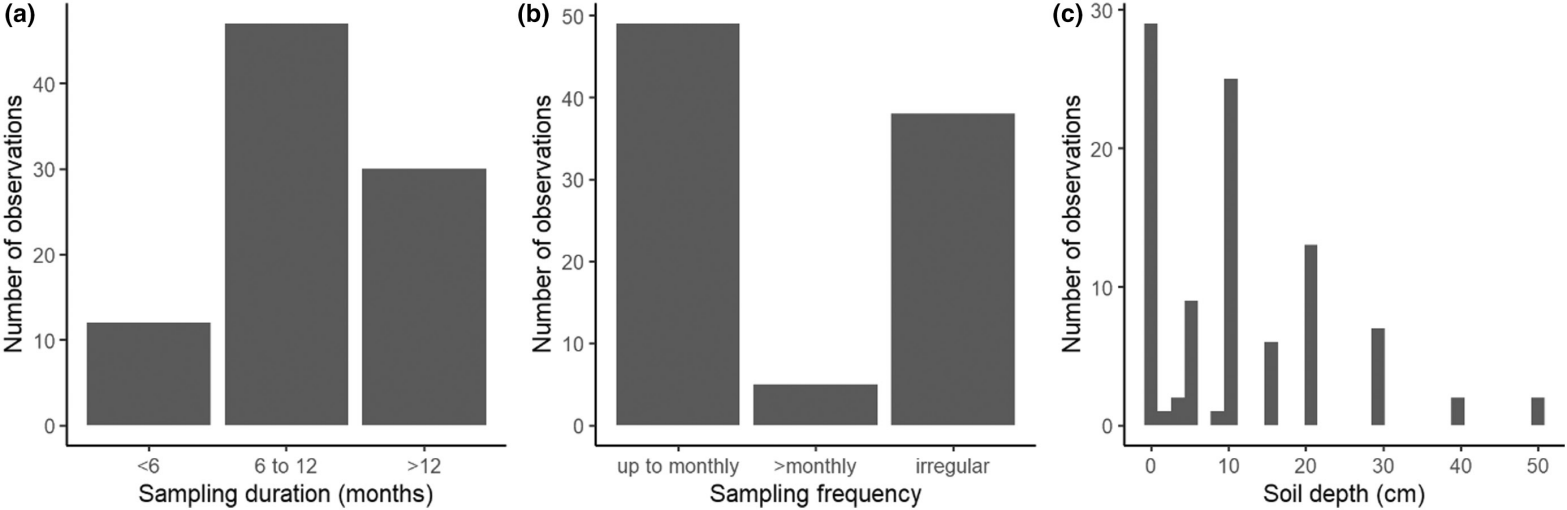
Methodological practices in soil phenology research. (a) The number of phenological observations by sampling duration. (b) The number of phenological observations by the sampling frequency. (c) The number of phenological observations conducted in different soil depths, from the litter layer (0 cm) down to a soil depth of 50 cm. The numbers on the x‐axis indicate the deepest sampled depth. If a sample was taken, e.g., at 50‐cm deep, and then divided into half for further analyses, one core was assigned at 25 cm and one at 50 cm.

One of the greatest challenges of synthesizing results is to evaluate results obtained from different methodological approaches (Kühl et al., 2020). This is especially critical in phenological studies when the sampling methods (e.g., duration and type, taxonomic group, and climate factors) may influence the interpretation of phenological data. Most studies on soil phenology have been conducted for six to 12 months (Figure 4a). The duration of aboveground phenology studies is often shorter and more easily distinguished since the timing of key life‐history events like the emergence of plant shoots and flowers are easily visible for days, weeks, or months. However, belowground organisms can remain active throughout the year. Consequently, there is substantial uncertainty about whether belowground organisms respond similarly to environmental conditions as plants. Indeed, there is evidence of decoupled activity between the above‐ and belowground plant parts (Abramoff & Finzi, 2015; Blume‐Werry et al., 2016; Schwieger et al., 2019). Also, some groups of soil animals, such as litter‐associated fauna, are known to peak in abundance, biomass, and activity at a different period than the peak of plant growth, such as ants in a tropical forest (Grimbacher et al., 2018) and deciduous invertebrates' larvae in a subtropical forest (Guo et al., 2020).

The sampling frequency is another critical aspect to be defined to capture finer or coarser changes. Many papers measured soil phenology below a monthly frequency (i.e., daily, weekly, biweekly), but the number of measures taken irregularly over the year was also prominent (Figure 4b). Such methodological decisions may stem from the fact that a big part of the studies considered in this review focused on gross changes across seasons. Although coarse temporal resolution sampling schemes may address the objectives of individual studies, researchers should be aware of the importance of taking measurements at a finer temporal scale to unravel more nuanced mechanisms driving soil phenological changes (Morellato, Camargo, et al., 2010).

Soil depth sampling is intrinsically associated with sampling frequency and duration, given their complementary roles in picturing soil biodiversity and functioning (Eisenhauer et al., 2018). Most of the studies covered only the shallow soil (first 10 cm, Figure 4c). This common practice is associated with the fact that most biological activity occurs in the first centimeters of the soil depth, even though biological activity may migrate to deeper soil layers as soil conditions in the top layers get harsh (Eisenhauer et al., 2021; White et al., 2020).

### 3.4 Summary of soil phenological data

After an overview of the biases in soil phenology research, we summarize the general patterns on how biotic and abiotic factors drive, or are associated with, the different types of phenological responses we assembled for both observational and experimental studies. Because we do not have enough data to quantitatively analyze phenological patterns, we qualitatively discuss the extent to which findings align with expectations for aboveground phenology or belowground ecology (Table 1). The microbiota abundances were mostly positively related to soil pH and N, and negatively to soil moisture and organic matter. For soil microbiota activity (i.e., soil respiration), experiments show a positive relationship between soil temperature and soil respiration, as well as SOM and P. The same pattern appears in observational studies, even though some findings show neutral or negative effects, possibly due to the wider diversity of ecosystem types and higher number of studies in observational settings. Moreover, the negative effects of temperature on soil microbiota activity could be due to a temperature‐induced reduction in soil moisture (Thakur et al., 2018). The effects of N on soil respiration are mixed, and positive, neutral, and negative effects are shown. Soil moisture also shows an inconsistent relation with soil respiration, with most studies detecting neutral effects. By contrast, microbiota abundance is positively related to soil moisture, organic matter, and N, while is negatively or not associated with soil temperature. The abundance of mesofauna (specifically mites) was negatively correlated with rainfall and soil temperature, which was associated with higher food availability and higher protection against predators provided by a denser litter layer during the dry season (Pequeno et al., 2017). Soil temperature also showed opposite effects on different macrofauna taxa—positive or neutral for arthropods and negative for earthworms, which is in line with what is expected for both groups. Additionally, earthworm biomass was positively correlated with soil moisture. In general, observational studies show more inconsistent patterns than experiments regarding the relationships between predictors and phenology. All biotic factors were positively associated with soil phenological responses. In general, microbiota shows a higher sensitivity to increased temperature and decreased soil moisture when compared to meso‐ and macrofauna, which could also be due to study bias towards more temperature‐resistant soil fauna groups. Including phenological biotic factors seems to be a good addition to providing new insights into what shapes soil organisms' phenology. However, caution is generally needed when trying to draw hard conclusions on this topic due to the low representation of taxa and ecosystems discussed in the previous sessions.

**TABLE 1:** Summary of evidence showing relationships between soil phenology and its predictors, in both experiments and observational studies.

Study type	Taxa		Phenology type	Predictors								
				Soil temperature	Rainfall	Organic matter	Moisture	N	P	pH	Abiotic factors (other)	Biotic factors
Experiment	Microbiota	Bacteria	Abundance					**↗** (3)		**↗** (1)		
		Archea					**↘** (2)			**↗** (2)		
		Fungi				**↘** (2)						
		–	Activity	**↗** (5, 6, 8, 9, 11, 12, 13, 14, 15)		**↗** (4)	**↘** (11, 12, 14) **→** (5, 6, 7, 8, 10, 12, 15)	**↘** (13) **→** (10) **↗** (6)	**↗** (6)			Root biomass **↗** (10); detrital mass **↗** (10)
		–	Biomass	**→ ↘** (17)		**↗** (17)	**↗** (16, 17)	**↗** (16)				
		Fungi	Diversity				**→** (20)	**↘** (20)		**→** (2, 18) **↗** (18, 20)		
		Nematodes						**↘** (19)		**↗** (19)		
		Archea								**→** (2)		
		Bacteria								**→** (2)		
Observation	Macrofauna	Arthropods	Abundance	**→** (21), **↗** (21, 22)	**→** (21, 23)						Air temperature **↘** (23) **↗** (48)	Potential prey abundance **↗** (23) seeding **↗** (57)
			Diversity	**↗** (21)	**→** (21)							
		Earthworm	Biomass	**↘** (29)			**↗** (29)					
			Abundance	**↗** (56)			**↘** (56)					
	Mesofauna	Arthropods	Abundance	**↘** (24)	**↘** (24)							Flowering **↗ ↘** (57)
	Microbiota	Bacteria	Abundance									Litter moisture **↗** (26)
		Nematodes				**↗** (27)	**↗** (27)					
		Fungi						**↘** (28)		**↘** (28)		
		–	Activity	**↗** (25, 29, 30, 33, 34, 35, 37, 39, 40, 42, 43, 44, 45, 54) **→** (31, 36, 37) **↘** (37, 41)	**→(**35) **↗** (31, 40)		**↗** (30, 31, 32, 34, 35, 36, 38, 40, 43, 44, 54) **→** (37) **↘** (25, 37, 41)	**↗** (31, 34, 37) **→** (37) **↘** (37, 43)		**→** (31) **↗** (29) **↘** (41)	Soil depth **↗** (55)	Leaf fall **↗** (39); root biomass **↗** (43, 44)
		–	Biomass	**↘** (46) **↗** (49)	**↘** (49) **↗** (49)		**→** (47) **↗** (47, 50)	**↗** (46)	**↗** (46)	**↗** (47) **→** (50) **↘** (49)		Detrital mass **↗** (46)
		Bacteria	Diversity	**↘** (53)		**↗** (53) **↘** (55)	**↗** (55)					
		Fungi		**→** (51)			**↗** (51)			**↗** (52)		
		Nematodes				**↗** (27)						

*Note*: Numbers indicate the sources of predictor–phenological response relationships and show the accumulation of positive (blue), neutral (yellow), and negative (red) relationships shown as arrows (see methods for description of calculations). Refer to Table S1 for a complete overview of the relationships upon which this table is based.

Sources: 1, Pereira e Silva et al. (2011); 2, Pereira e Silva et al. (2012); 3, Wang et al. (2016); 4, Carranca et al. (2009); 5, Dacal et al. (2020); 6, Fan et al. (2015); 7, Francioni et al. (2020); 8, Goncharova et al. (2020); 9, Han & Jin (2018); 10, Han et al. (2007); 11, Han et al. (2016); 12, Han et al. (2017); 13, Peng et al. (2019); 14, Pries et al. (2020); 15, Zhang, Qian et al. (2020); 16, Chen et al. (2020); 17, Shahbaz et al.  (2020); 18, Bainard et al. (2014); 19, Song et al. (2015); 20, Wei et al. (2020); 21, Ahrens et al. (2009); 22, Kostromytska and Buss (2008); 23, Medeiros et al. (2012); 24, Pequeno et al. (2017); 25, Barba et al. (2018); 26, Krivtsov et al. (2005); 27, Pen‐Mouratov and Steinberger (2005); 28, Zhang, Zhou et al. (2020); 29, Bayranvand et al. (2017); 30, Butler et al. (2012); 31, Chen et al. (2010); 32, Cusack et al. (2019); 33, Davidson et al. (1998); 34, Fernandez et al. (2006); 35, Gömöryová et al. (2013); 36, Knowles et al. (2015); 37, Lo Cascio et al. (2017); 38, Meena et al. (2020); 39, Raich (2017); 40, Rochette et al. (1991); 41, Shi et al. (2020); 42, Xiao et al. (2014); 43, Zhou & Zhang (2014); 44, Zhu et al. (2019); 45, Zimmermann et al. (2010); 46, Barbhuiya et al. (2004); 47, Baum et al. (2003); 48, Oliveira et al. (2009); 49, Patel et al. (2010); 50, Wang et al. (2020); 51, Burke et al. (2019); 52, He et al. (2017); 53, Wang et al. (2018); 54, Cui et al. (2020); 55, Cartwright and Hui (2015); 56, Eggleton et al. (2009); 57, Kwok et al. (2016).

## 4 GOOD PRACTICE IN SOIL PHENOLOGY RESEARCH: RECOMMENDATIONS AND HIGHLIGHTS

Although many of the recommendations for good practice in measuring soil phenology rely on general recommendations for soil measurements, some recommendations are particularly pertinent for the analysis and interpretation of temporal patterns. This section highlights papers that have explored promising, albeit mostly underexplored, hypotheses and showed good practice with regard to either methodological approaches or reporting data and results.

### 4.1 Emerging research foci

The first experiments on the effects of climate change on belowground communities have been emerging only in the last few years. For instance, Thakur et al. (2018) and Sünnemann et al. (2021) found that warming and altered rainfall patterns decrease invertebrate decomposer activity. Siebert et al. (2019) found similar results for the magnitude of soil biological activity under elevated temperature and shifted precipitation patterns, adding that a more extensive land‐use regime could not buffer future climate effects. They also found that the phenological peaks of soil activity were advanced in around 15 days in habitats subjected to experimental warming (Siebert et al., 2019). Such results represent a great advance in understanding how warming affects decomposition, a key aspect of the well‐being of ecosystems (Swift et al., 1979). Future studies should also examine other taxa and ecosystems since bigger organisms with slower metabolism may tolerate the increase and the variation in temperature differently (LaBarbera, 1989), which does not allow us to have a clear perception of whether and how soil phenology is affected by global change.

Among the studies that consider biotic factors as predictor variables, litter‐related factors are among the most prevalent ones, which seems logical given the close relationship between the litter layer and soil organisms (Pollierer et al., 2007; Ruf et al., 2006). However, the direction and magnitude of results have been mixed, suggesting that more research is needed to resolve the nature of these relationships. For example, peaks of litter biomass and increases in litter diversity have been shown to be positively related to peaks of microbial biomass (Barbhuiya et al., 2004; Chemidlin Prevost‐Boure et al., 2011) and abundance (Krivtsov et al., 2005), respectively. However, Doblas‐Miranda et al. (2007, 2009) found that litter production and detritivore abundance were negatively correlated in a desert ecosystem because the peak of litter production is in the summer when a water deficit restricts litter palatability. Guo et al. (2020) investigated the relationship between the changes in litter decomposability during the decomposition process with invertebrate phenology to understand how these two factors combined affect litter decomposition in a subtropical forest. They found that during the later stages of litter decomposition when most of the litter is recalcitrant matter, the increased activity of decomposers was essential to mass loss. Those findings highlight that although litter quality and litter quantity alone are crucial predictors of litter decomposition, this relationship may be strongly modulated by both plant and soil phenology and climatic seasonality, even leading to counterintuitive results.

### 4.2 Key methodological considerations

Methodological decisions, such as sampling duration and frequency, are critical in phenological studies since they can determine how well the activity periods of target organisms can be detected. Ideally, the pace of measurements should be determined by the biology of the focal group and response variable reported. For example, vegetative organs of evergreen trees can be monitored using monthly measurements (Morellato, Camargo, et al., 2010; Perina et al., 2019), but biweekly, weekly, or daily measurements are needed to capture finer‐scale variation of flowering and fruiting phenophases in seasonal grasslands (Morellato, Camargo, et al., 2010). Similarly, the frequency of soil measurements should increase, e.g., from higher than monthly to monthly or biweekly, when dealing with more sensitive organisms (i.e., microbiota and small invertebrates) or functions (e.g., soil respiration), so that key phenological events can be detected with greater accuracy.

Climatic factors influence soil organisms and functions at different intensities and depths (Eisenhauer et al., 2018). For example, while diversity is often concentrated in the top layers of tropical soils, thick organic layers may harbor more soil biodiversity and activity in deeper soil layers in harsh environments, such as boreal ecosystems and deserts (hot and cold) (Palm et al., 2007; Ross, 1993). Also, because climatic conditions change over the year, vertical migration may play an essential role in soil functioning, with the deepest layers harboring soil activity during harsh climatic conditions and extreme events, such as frost or drought. Seeking evidence of vertical stratification of soil activity, Doblas‐Miranda et al. (2007) conducted a study in a Mediterranean desert and sampled soil up to 50 cm in depth. They showed that both upper and lower soil layers were affected by climate seasonality, but soil community aspects, measured by richness, abundance, similarity, and biomass, were generally higher in the deeper layers (Doblas‐Miranda et al., 2007). Therefore, sampling in different depths (as suggested by White et al., 2020), combined with multiple sampling methods, allows researchers to capture organisms with different feeding habits, enabling the calculation of matter and energy fluxes throughout a comprehensive food web over time (see Eisenhauer et al., 2021 for a deeper discussion on the accountancy of repeated measures of soil organisms and functions).

While some ecosystems, communities, and populations worldwide show a marked phenological peak, others behave more smoothly, as a continuum with no abrupt transitions (Sakai, 2001; Staggemeier et al., 2018). Plant phenology may respond to climate through trigger thresholds (e.g., after a given temperature is surpassed, a physiological program unwinds), while the soil may respond continuously to climatic conditions. This requires that statistical approaches are able to embrace and compare those differences accurately. One way to accomplish that is by using circular statistics that account for the phenological structure by using the angles of a circle as response variables, while trigonometric functions and cartesian coordinates are used to calculate further statistics, such as circular mean and circular standard deviation (Morellato, Alberti, & Hudson, 2010). Also, the fit of Generalized Additive Mixed Models (GAMM) with cyclic/circular splines or sine‐cosine transformations (Polansky & Robbins, 2013) may be helpful. However, even in classic plant phenology research, the circular nature of phenological data has been ignored, which might lead to misestimates of, for example, the peak date (Staggemeier et al., 2018). The same issue may appear when dealing with soil phenology. Specifically, larger soil animals, such as macroinvertebrates or some fungi (e.g., basidiomycetes), may exhibit a peaked temporal pattern with a clear resting period during the winter in temperate regions while showing a continuous, low seasonal pattern in tropical, nonresting systems. In this case, the strong seasonal phenology could be accurately analyzed through both linear and circular approaches, but the less seasonal phenology can show large differences in statistical estimates between both approaches.

### 4.3 Reporting phenological results

Classic plant phenology papers are used to show ombrothermic diagrams (also known as Walter's climate diagrams) to describe the study area climatically. Such graphs are constructed with three variables: time on the x‐axis, and rainfall and temperature are often shown on the y‐axis (Engel & Martins, 2005). Although recent plant phenology research has been slowly abandoning such graphs, probably due to the increase in big data sets, they can be very informative and handy at helping to identify important events for phenological patterns, such as water deficit and excess periods, due to the different scaling of the two y‐axes. Some studies report an ombrothermic diagram for soil phenology (see Legakis & Adamopoulou, 2005). In all cases, its presence allows the evaluation of correlations between phenological events and climatic changes. However, soil structure often mediates effects on the soil microclimate. For example, Palm et al. (2007) show that sandy soils have a shorter memory of rainfall events than loess soils because of the difference in permeability. Therefore, when dealing with soil organisms, measures directly taken from the soil, such as soil temperature and moisture, might be a better choice, given that those parameters are more direct drivers of microbial activity than atmospheric temperature and precipitation. Indeed, previous work has shown distinct global mismatches between soil and air temperature (Lembrechts et al., 2022), and data availability on soil temperature is constantly increasing (Lembrechts et al., 2020).

## 5 GAPS, CHALLENGES, AND OPPORTUNITIES

To move towards a more comprehensive understanding of soil phenology, this research needs to consider some aspects. First, clear phenology indicators or metrics, such as phenological duration, onset, or offset, are needed to document phenological events that could be comparable among places to find consistent measures of phenological change. Second, since phenological shifts of a few days are hardly noticeable with few samplings per year, samplings at finer temporal resolution (less than monthly, when possible) are needed if the goal is to detect the effects of global change.

One crucial aspect of global change is biodiversity change, which has led to an increasing number of studies accounting for diversity as an important driver of ecosystem functioning in the last three decades (Cardinale et al., 2012; Hines et al., 2019). The effects of diversity on ecosystem functions and processes are explained by two main mechanisms, one capturing species niche differences and facilitation (i.e., complementarity) and the other accounting for the inclusion of particularly well‐performing species at a given location (i.e., sampling or selection) (Hooper et al., 2005; Loreau & Hector, 2001). Although they are classically studied concerning the spatial scale (i.e., spatial resource partitioning), these biodiversity mechanisms can easily apply to phenology (i.e., temporal resource partitioning; Ebeling et al., 2014). Suppose the temporal axis of a species' ecological niche can be measured based on its timing of activity. In that case, the degree of temporal specialization or generalization can be calculated, as well as the degree of temporal overlap with other species in the community. Guimarães‐Steinicke et al. (2019) showed that the start and end of plant productivity in a grassland tended to be controlled by plants with early and late phenology, respectively. This suggests that increasing phenological richness can temporarily extend and potentially increase the annual biomass production, but other works have found no significant effects of phenological complementarity on ecosystem functioning (Henry et al., 2001; Zhao et al., 2007). Moreover, it can be suggested that biodiversity may act on community temporal patterns through species selection; increasing diversity may increase the chance of having a temporally dominant species (or temporally generalist, which can occur under different climatic conditions according to the degree of competition in the “hot moments”), that extends the overall activity of the community. In fact, there is first empirical evidence that diversity in both spatial and temporal resource partitioning can influence soil organisms (Eisenhauer, 2016).

Plant‐related drivers (e.g., plant biomass, plant diversity, plant litter biomass) were the most common biotic predictors accounted for in soil phenology research (Table S1). Although they are often measured once, with no attribution to soil phenological patterns, plants buffer soil temperature (Guimarães‐Steinicke et al., 2021), which may directly influence soil activity. In light of this, we suggest attributing plant phenology as a driver of soil phenology would allow mechanistic assessments of plant effects over the year through plant–soil feedbacks and coupling of aboveground‐belowground activities. Other biotic factors regarding species interactions, specifically trophic interactions, may be critical in some ecosystems. For example, herbivores' presence, density, and activity appear to drive changes in plant quality, act as selective agents of plant community composition, and increase root exudation (Bardgett & Wardle, 2003). Larger herbivores, such as cattle, horses, and goats, may also substantially increase the nutrient input to the soil through dung and urine (Andriuzzi & Wall, 2017; Bardgett & Wardle, 2003). Additionally, the magnitude and direction of these effects seem to depend on the climate, herbivore identity, ecosystem type, and focal soil function and process (Andriuzzi & Wall, 2017). These effects may reveal a seasonal pattern in nutrient availability and the peaks of root exudation, microbial activity, and nutrient mineralization, especially in ecosystems seasonally used by animals during migrations events (e.g., bovines taken to a higher land during seasonal floods or natural migration of large herbivores during the winter).

Derived from the overall geographical biases in science, soil phenology research has been primarily conducted in subtropical and temperate environments. Mellard et al. (2019) conducted a review of phenological diversity patterns among biomes, and, besides, they found seasonal patterns in all regions, the overall magnitude, variation, and peak periods of diversity differed substantially between terrestrial temperate and tropical ecosystems. In addition to that, the Intergovernmental Panel on Climate Change (IPCC, https://www.ipcc.ch/) foresees an increase in the average temperature and climate variability. Altogether, this evidence highlights the need to extend future research of soil phenology to hot seasonal environments, allowing us to understand how natural ecosystems will behave in future, allowing us to make better management and political decisions.

Ecological knowledge generated and maintained by traditional groups may provide important guidance in places with significant academic knowledge gaps (Albuquerque et al., 2021). For plant phenology, the information provided by traditional people was as accurate as data collected by academic scientists (Campos et al., 2018; Lins Neto et al., 2013). When applied to the soil, traditional ecological knowledge may provide substantial information on which biotic and abiotic factors guide the presence and absence of specific soil organisms, point out the reproductive periods of species, and describe the importance of organisms to determine ecosystem functioning. Related to that, the engagement of nonscientists in the scientific process via citizen science may also help to identify phenological trends for bigger soil organisms from local to global scales. The use of citizen science has been increasing in recent years, especially focusing on biodiversity monitoring, with successful cases such as the Urubu System for road monitoring (https://sistemaurubu.com.br/), the iNaturalist (https://www.inaturalist.org/), or the PhenObs (Nordt et al., 2021), which are already focused on plant phenology. First identification apps and citizen science projects are also starting for soil organisms (https://bodentierhochvier.de/en/soil‐animal‐portal‐for‐experiencing‐identification‐recording‐and‐exploring/) and could be extended to phenological assessments. These could be used to track one or a few species, selected by local specialists, that are highly correlated with key climatic variables (e.g., temperature or rainfall) to determine their onset, duration, and decline dates. Therefore, we could compare those dates among different years and detect phenological shifts in soil organisms. With a combination of information provided by citizens (e.g., pictures, date, and place) and specialists (e.g., taxonomic identity), we could identify phenological peaks, absences, interactions among species, and correlations with biotic and abiotic factors.

Another opportunity to balance research inequalities between the Global North and South is by creating global networks in which scientists share funding and staff (Maestre & Eisenhauer, 2019). However, given the high number and/or high quality of both phenological and soil networks worldwide, creating a collaborative network focused on soil phenology does not seem to be necessary. Instead, we advocate that existing phenological networks, primarily focused on plant phenology, could also benefit from soil measurements and standard sampling methodology (Bruns et al., 2003). Such existing networks could be global as the Global Phenological Monitoring, the Global Soil Biodiversity Observation Network (SoilBON), and Nature's Calendar, or countrywide, such as the National Phenological Network in the United States, the Observatoire des Saisons in France, the e‐phenology in Brazil, and the Chinese Phenological Observation Network.

Understanding why and how populations and communities change over the year is a significant step towards a broader knowledge of ecosystem functioning. Soil organisms' phenology is a relatively undeveloped research area, with substantial geographical and taxonomic biases to overcome before it can be used to guide effective conservation and management decisions. Because soil encompasses a great diversity of organism sizes and functions, there may be a significant variation of phenological responses to the same driver (Blankinship et al., 2011; Siebert et al., 2019), increasing the chance of temporal complementarity among soil organisms and uncoupling with aboveground phenology. Therefore, we advise that researchers adjust the sampling frequency according to the local climatic, focused taxa, and phenology types for adequate sampling, covering the entire year if possible. At the same time, we suggest creating a generalized protocol advising minimum requirements of sampling standardization for future synthesis purposes. We should also advance on metrics for soil phenology that could be comparable among ecosystems. Biotic drivers, such as above‐ or belowground diversity and structure, are rarely accounted for in soil phenology research. Such knowledge, however, will be increasingly important in our changing world since biodiversity loss and community change are crucial consequences of global change. We advocate that current experiments on biodiversity and ecosystem functioning, as well as plant phenology projects, could be extended to incorporate phenological measurements of soil, helping to overcome this knowledge gap.

## AUTHOR CONTRIBUTIONS

**Ana E. Bonato Asato:** Conceptualization (equal); data curation (lead); formal analysis (lead); investigation (lead); methodology (lead); software (lead); validation (lead); visualization (lead); writing – original draft (lead); writing – review and editing (equal). **Christian Wirth:** Funding acquisition (equal); investigation (supporting); project administration (equal); resources (equal); writing – review and editing (equal). **Nico Eisenhauer:** Conceptualization (equal); funding acquisition (equal); methodology (supporting); project administration (equal); resources (equal); supervision (lead); validation (equal); writing – original draft (supporting); writing – review and editing (equal). **Jes Hines:** Conceptualization (equal); data curation (supporting); formal analysis (supporting); funding acquisition (equal); investigation (supporting); methodology (supporting); project administration (equal); resources (supporting); supervision (lead); validation (lead); visualization (supporting); writing – original draft (supporting); writing – review and editing (equal).

## ACKNOWLEDGEMENTS

We thank Justus Hennecke for the assistance with the data extraction and Olga Ferlian for advice on search terms definition and the paper's inclusion criteria. We also thank the anonymous reviewers for the valuable and kind comments, which have helped enormously the improvement of this manuscript.

## FUNDING INFORMATION

We acknowledge the support of iDiv funded by the German Research Foundation (DFG–FZT 118, 202548816). The Jena Experiment is funded by the DFG (FOR 5000).

## Supporting information


Table S1.

## Data Availability

The data that support the findings of this study are available in the supplementary material of this article.
